# Alterations in Soluble Class III Peroxidases of Maize Shoots by Flooding Stress

**DOI:** 10.3390/proteomes2030303

**Published:** 2014-06-26

**Authors:** Claudia-Nicole Meisrimler, Friedrich Buck, Sabine Lüthje

**Affiliations:** 1 University of Hamburg, Biocenter Klein Flottbek and Botanical Garden, Oxidative Stress and Plant Proteomics Group, Ohnhorststraße 18, D-22609 Hamburg, Germany; E-Mail: c_m_2406@yahoo.de; 2 University Hospital Eppendorf, Institute of Clinical Chemistry, Campus Science, Martinistraße 52, D-20246 Hamburg, Germany; E-Mail: buck@uke.de

**Keywords:** flooding, water logging, guaiacol peroxidases, native PAGE, soluble proteins, shoot, *Zea mays* L.

## Abstract

Due to changing climate, flooding (waterlogged soils and submergence) becomes a major problem in agriculture and crop production. In the present study, the effect of waterlogging was investigated on peroxidases of maize (*Zea mays* L.) leaves. The plants showed typical adaptations to flooding stress, *i.e.*, alterations in chlorophyll *a/b* ratios and increased basal shoot diameter. Seven peroxidase bands could be detected by first dimension modified SDS-PAGE and 10 bands by first dimension high resolution Clear Native Electrophoresis that altered in dependence on plant development and time of waterlogging. Native isoelectric focusing revealed three acidic to neutral and four alkaline guaiacol peroxidases that could be further separated by high resolution Clear Native Electrophorese in the second dimension. One neutral peroxidase (pI 7.0) appeared to be down-regulated within four hours after flooding, whereas alkaline peroxidases (pI 9.2, 8.0 and 7.8) were up-regulated after 28 or 52 h. Second dimensions revealed molecular masses of 133 kDa and 85 kDa for peroxidases at pI 8.0 and 7.8, respectively. Size exclusion chromatography revealed native molecular masses of 30–58 kDa for peroxidases identified as class III peroxidases and ascorbate peroxidases by mass spectrometry. Possible functions of these peroxidases in flooding stress will be discussed.

## 1. Introduction

Weather records documented a steady and significant increase in flooding events over the past six decades [1]. As a consequence, crop fields are more often overflooded by extreme water levels of rivers and heavy rain falls. Survival of plants under those conditions depends on physiological, morphological and metabolic adaptations [2].

Depending upon the moisture or water level on the field, flood, submergence or soil saturation, can be distinguished for waterlogging. Two types of flooding are generally discriminated in the field: (1) waterlogging, in which root and some portion of the shoot are under water or the soil appears water saturated without free-standing water; and (2) complete submergence, where the whole plant is under water [3].

Waterlogged soils provoke iron toxicity and low oxygen levels in roots. Oxygen levels are characterized by two terms: (1) Hypoxia, reduction of oxygen below optimal levels and (2) anoxia, the complete lack of oxygen, which occurs in soils that are exposed to long-term flooding and complete submerging [4].

Waterlogging resistant plants like maize (*Zea mays* L.) adapt to waterlogged conditions by developing aerenchyma in roots for ventilation and some wetland plant species form an apoplastic barrier at the outer cell layers of roots to reduce radial oxygen loss [5]. The apoplastic barrier composition is not well understood, but one potential component is suberin, which accumulates at the hypodermal/exodermal cell layers of the roots under waterlogged soil conditions. However, variation between plant species makes evaluation of the significance of suberin in prevention of radial oxygen loss rather difficult.

Depending on flooding conditions—short-term (<two weeks) or long-term submergence—plants evolved two different strategies [6]. Plants temporally flooded like maize or tufted hairgrass (*Deschampsia cespitosa* L.) show low oxygen quiescence or avoidance syndrome, whereas species like deep water rice (*Oryza sativa* L.) showed low oxygen escape syndrome [7,8,9]. One of the key players in rice and wetland species grown under submerged conditions is ethylene, which induces (i) aerenchyma in the root cortex by programmed cell death; (ii) adventitious root growth and (iii) elongation of internode by regulation of gibberellic acid biosynthesis and sensitivity [3]. These adaptations provide leaf contact with the atmosphere under submerged conditions and enhanced gas diffusion. The molecular mechanisms induced by flooding have been intensively investigated in deep water rice and *SNORKEL* genes that encode transcription factors of the AP2/ethylene response factor (ERF) family subgroup VII have been discovered [7]. In contrast to the low oxygen escape syndrome, plant species confronted with short-term flooding stress (partial or complete submergence) maintained steady energy conservation without shoot elongation [8]. It is known that another member of the AP2/ERF family mediates the quiescence syndrome (SUB1A) [10].

Aside, nutrient uptake and photosynthesis are affected by flooding in general and changes in chlorophyll a/b ratios in the foliage were observed in both cases [11,12,13]. Furthermore, the role of reactive oxygen species (ROS) has been discussed recently for both stresses [5,14]. Low photon utilisation of flooded plants could result in the production of ROS like superoxide anion radicals, singlet oxygen, hydrogen peroxides and hydroxyl radicals [15]. These ROS are very reactive and provoke damage to lipid membranes and proteins. To manage the level of ROS plants have antioxidants (e.g., ascorbate, glutathione and tocopherols) and ROS scavenging enzymes like superoxide dismutase or peroxidases [16]. Peroxidase activity is used as a general stress marker. Class III peroxidases (secretory pathway) are antioxidative systems involved in several physiological functions including plant development, cell wall related processes and oxidative stress [17,18,19,20]. Due to their reactive cycles, heme-containing peroxidases are involved in both production and detoxification of ROS and are affected under several stress conditions [19,20,21]. Cytosolic ascorbate peroxidase of soybean (*Glycine max* L.), a flooding sensitive plant species, decreased under submerged conditions [22,23]. In contrast to this observation, peroxidase activity increased in flooding tolerant clover and an additional isoperoxidase was induced [24]. Although total peroxidase activity of plant extracts is a stress marker, results are not clear, because of the high amount of isoenzymes that may be differentially regulated [25]. To distinguish between several isoenzymes and to identify peroxidases involved in a specific stress response, proteomic approaches are state of the art [25,26]. Additionally, advantages of proteomic approaches in studying flooding have been summarised [27]. Protocols for separation of class III peroxidases by native and in-native 2D-PAGE and detection by peroxidase specific in-gel stains have been published by our team [26,28].

Besides rice, grasses like barley (*Hordeum vulgare* L.), wheat (*Triticum aestivum* L.) and maize belong to the flooding tolerant plants. Although maize is one of the most important crop plants in agriculture and biochemical studies indicate its flooding tolerance [27], proteomic approaches have not been presented for leaves of waterlogged maize. In the present study, profiles of soluble proteins were analyzed from leaves of control and waterlogged maize plants that showed typical stress symptoms. Alterations in profiles of class III peroxidases were investigated by modified SDS-PAGE and native isoelectric focusing (IEF) combined with guaiacol staining. IEF-gels were transferred to second dimension modified SDS-PAGE or high resolution Clear Native Electrophoresis (hrCNE) for further separation of isoperoxidases. Possible functions of identified peroxidases in flooding stress will be further discussed in the results and discussion section.

## 2. Experimental

### 2.1. Plant Material

Maize plants (*Zea mays* L. cv. Gelber Badischer Landmais, Saatenunion, Hannover, Germany) were grown in the green house (28 °C at day; 16–18 °C at night; 1000 µmol/m^2^·s ± 50 µmol/m^2^·s) for 28 days on potting soil. At day 29, plants were flooded continuously. Flooding conditions were done without additional oxygen supply. Oxygen concentration, pH and water temperature were checked constantly. Water temperature was steadily 20 °C ± 0.5 °C and pH was 5.6 for all three time points. The water level was held at 15 cm above the soil surface. The control plants were kept in soil without flooding and were continuously watered indirectly from the bottom and once per day from the top. Water content was held between 20% and 30%. All leaves of the shoot were harvested from control and flooded plants 4 h, 28 h and 52 h after induction of flooding. Samples were taken always at the same time point of the day and for each condition four pools containing five biological replicates were collected. Shoot length was determined for each time point using the same 20 plants.

Statistics and diagrams were calculated using OriginPro 8.5.1.G (Additive GmbH, Friedrichsdorf, Germany). For all measurements, standard deviation was calculated and student’s *t*-test was used to determine the significance of changes (control *versus* stressed sample).

### 2.2. Determination of Chlorophyll Concentrations

Leaves were grinded with liquid nitrogen before chlorophyll was extracted using a 90% acetone solution. After extraction for 30 min in the dark, extract was filtered and volume was made up to 50 mL. Chlorophyll *a* and chlorophyll *b* concentrations were determined spectrophotometrically, using the absorption maximum at 663 nm for chlorophyll a and the maximum at 646 nm for chlorophyll b [29]. Based on the absorption, chlorophyll concentration per g fresh weight was calculated for control and stressed plants.

### 2.3. Protein Extraction

Soluble proteins of shoots were separated from the microsomal proteins by differential centrifugation as described elsewhere [30]. Soluble proteins were concentrated and desalted using spin columns (Millipore, MWC 10,000, Schwalbach, Germany) and protein amounts were quantified as described by Bradford [31] using bovine serum albumin as standard. Samples were stored at −76 °C until further use.

### 2.4. Size Exclusion Chromatography

Proteins were separated by size exclusion using an HPLC-System (ÄKTA, Amersham Pharmacia Biotech, Freiburg, Germany) with a 2-mL loop. All steps were performed at 4 °C. Samples were concentrated (Centricon YM-10 concentrators; Millipore, Bedford, MA, USA). Concentrated fractions (40–60 µL) or calibration proteins (thyroglobulin (669 kDa), ferritin (440 kDa), catalase (232 kDa), aldolase (158 kDa), bovine serum albumin (68 kDa), horseradish peroxidase (44 kDa), and ribonuclease A (13.7 kDa), Amersham Pharmacia Biotech) were applied on a Superdex 200 column (HR 10/30, GE Healthcare) equilibrated with four column volumes of phosphate buffer (50 mM Na_3_PO_4_ (pH 7.0), 150 mM NaCl, 1 mM CHAPS, 1 mM EDTA and 1 mM ascorbate). Proteins were eluted by 1.5 column volumes of buffer. The flow rate was 0.5 mL min^−1^. The fraction size was 0.5 mL. Peroxidase containing fractions were identified by a microassay in 96 well plates. The assay contained 20 µL protein fraction, 180 µL 50 mM Na-acetate buffer, pH 5.5, 25 µL guaiacol (826 mM) and H_2_O_2_ (8.8 mM) each. Estimates of the molecular masses of peroxidases were calculated using a semi-logarithmic plot of the molecular mass values for the calibration proteins against the elution volumes. For each sample, three biological replicates have been separated.

### 2.5. Gel Electrophoresis

One dimensional modified SDS-PAGE (12% acrylamide, no reducing agents, no heating of the samples), native IEF-PAGE and hrCNE were used for separation of soluble proteins. Electrophoresis of modified SDS-PAGE was done at 200 V and 4 °C. First dimensions IEF was accomplished in a mini gel cell (Biorad, Munich, Germany). Gels (0.075 × 7 × 8 cm) contained 4 M urea, 2% 3-[(3-cholamidopropyl)dimethylammonio]-1-propanesulfonate (CHAPS), 2% carrier ampholytes pH 3–10 (Serva, Heidelberg, Germany) and 5% acrylamide. Electrophoresis was carried out for 120 min at 100 V, 90 min at 250 V and 30 min at 350 V at 4 °C with 10 mM phosphoric acid and 20 mM NaOH as respectively anode and cathode buffer [28]. Isoelectric points were calculated in comparison with the pH of gel segments derived from control lanes. IEF-PAGE in the first dimension was followed by activity in-gel staining or by the second dimension modified SDS-PAGE and hrCNE). First dimension hrCNE was casted as continues gradient gel (6%–15% acrylamide concentration), electrophoresis was conducted for 45 min at 100 V, followed by 500 V and restriction to 10 mA per gel until the ponceau S reached the bottom of the gel. Gel lanes of the first dimension were equilibrated in loading buffer for modified SDS-PAGE (125 mM Tris-HCl, 0.2% (*w*/*v*) SDS, 20% (*w*/*v*) glycerol, and 0.004% (*w*/*v*) bromo-phenol, pH 6.8) or hrCNE (75 mM imidazole, 1.5 M 6-aminohexanoic acid (ACA), 0.03% Na-deoxycholate and 0.004% ponceau S pH 7.0) for 20 min at room temperature and applied to the second dimension modified SDS-PAGE or hrCNE [21,23]. Second dimension modified SDS-PAGE and hrCNE were performed similar to the first dimension described above. Gels were stained directly with guaiacol/H_2_O_2_ after the electrophoresis was finished. After 5 min of activity staining, gels were scanned as TIF-file for documentation (400 DPI, Perfection V700 Photo, EPSON GmbH, Meerbusch, Germany). Prestained marker (Fermentas, St. Leon-Rot, Germany) was used as a standard for all SDS-PAGEs. Gels used for guaiacol staining were generally loaded with 40 µg soluble proteins, except for proteins loaded on first dimension hrCNE (25 µg) used for the calculation of native molecular mass of peroxidases. Fractions, resulting from the separation by SEC, tested positive for guaiacol activity in the micro assay were also analysed by one dimensional PAGE. For each active fraction, 25 µL were mixed with the PAGE corresponding sample buffer and loaded on the gel.

### 2.6. Peroxidase Detection

In-gel peroxidase staining was accomplished with guaiacol/H_2_O_2_ (1:1) in 50 mM Na-acetate buffer pH 5.0, containing 10 mM CaCl_2_ [28].

### 2.7. Protein Digestion

The gel bands were cut out, the proteins reduced with DTT (10 mM, 56 °C, 30 min.), the cysteine residues modified with iodoacetamide (55 mM, ambient temperature, 20 min. in the dark) and the protein in-gel digested with trypsin (conditions: 5 ng trypsin/µL (sequencing grade modified trypsin, Promega, Madison, WI, USA) in 50 mM NH_4_HCO_3_, 37 °C, 16 h).

After digestion, the gel pieces were repeatedly extracted (50% acetonitrile/5% formic acid) and the combined extracts dried down in a vacuum concentrator.

### 2.8. Mass Spectrometry

For QTOF, Premier tandem MS analysis peptide extracts were dried down in a vacuum concentrator and resuspended in 20 mL 0.1% formic acid. The samples were centrifuged at maximal speed in an Eppendorf centrifuge and 2–4 μL of the digest used for an LC-MS run. LC-MS runs were done on a QTOF Premier tandem mass spectrometer (Waters-Micromass, Eschborn, Germany) equipped with an Aquity UPLC (Waters, Germany). Samples were applied onto a trapping column (Waters nanoAquity UPLC column, C18, 180 μm × 20 mm), washed for 10 min with 5% acetonitrile, 0.1% formic acid (5 μL/min) and then eluted onto the separation column (Waters nanoAquity UPLC column, C18, 1.7 μm BEH130, 75 μm × 200 mm, 200 nL/min) with a gradient (A, 0.1% formic acid; B, 0.1% formic acid in acetonitrile, 5%–50% B in either 60 or 120 min). The spray was done from a silica emitter with a 10 μm tip (PicoTip FS360-20-10, New Objective) at a capillary voltage of 1.5 kV. For data acquisition the MS^E^technique was applied: alternating scans (0.95 s, 0.05 s interscan delay) with low (4 eV) and high (ramp from 20–35 eV) collision energy was recorded [32,33]. The data were evaluated with the software package Protein Lynx Global Server version 2.3 (Waters) searching the Uniprot database (Jan 2014 update) and the Peroxibase. At intervals of 10 s, a lockspray spectrum (1 pmol/μL [Glu1] Fibrinopeptide B (Sigma)) was recorded. Using lockspray correction a mass accuracy of <7 ppm was achieved in the MS mode.

## 3. Results and Discussion

Additional information on the results can be found in the Supplemental data. The change of specific physiological parameters, morphological and anatomical changes have been shown to be typical for flooding stress. Chlorophyll a/b ratio and changes in the morphology were used to prove that plants showed typical changes in these parameters. These parameters can be often seen in context with the level of ROS, like superoxide anion radical, singlet oxygen, hydrogen peroxide and hydroxyl radical, which are highly reactive. They can provoke damage to various molecules, therefore they are tightly managed to protect cells against oxidative stress. It is known that peroxidases are part of this ROS scavenging mechanism; therefore, it is important to understand their regulations under flooding stress. Additionally, they play a role in cell wall loosening and reorganisation, such as needed for the formation of aerenchyma. Peroxidases were studied on two levels: (1) The abundance was studied by modified SDS-PAGE and (2) changes in activity were studied by native PAGE. Both methods were combined with guaiacol peroxidase specific in-gel staining and identification was done by LC-MS.

### 3.1. Physiological Parameters

**Figure 1:**
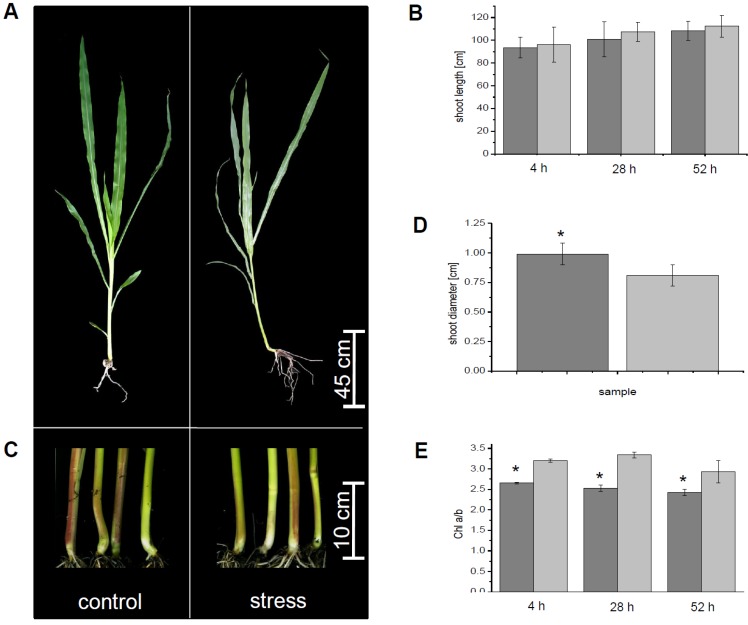
Morphological adaption of maize to submergence. (**a**) Phenotype of control plants and plants stressed by submergence at the end of the growing period; (**b**) Shoot length was measured for control and stressed plants over the three days of flooding; (**c**) Shoot basis of control and submerged plants; (**d**) Comparison of basal shoots of control and stressed samples at the end of the experiment; (**e**) Chlorophyll a/b ratio. All measurements were done for *n* ≥ 20 biological replicates. Except for (**e**) measurements were done for *n* ≥ 5 biological replicates. Control, light grey, flooded plants, dark grey. Significant changes were marked with an asterisk.

After flooding, the plants oxygen concentrations in the water were measured continuously because, as with waterlogging stress in nature, no adjustments were done to change oxygen concentration in one direction or another. After four hours of waterlogging, oxygen concentration was 84%, after 28 h it decreased to 22% and on the last measurement point, after 52 h, it was down to 2%. Typical symptoms of flooding stress were found for maize after three days of waterlogging. The roots of flooded plants tended to become negatively gravitropic (Figure 1A). Shoot length was compared for control and flooded plants at four, 28 and 52 h. The ratio of the average shoot growth (4 h: 1.0, 28 h: 1.0 and 52 h: 1.1) of control *versus* submerged plants did not change significantly (Figure 1B), but showed already a tendency of a decreased growth of the stressed plants. Furthermore, after 52 h of flooding stress, the shoot stem diameter was increased about 24% within stressed plants in comparison to the control plants (Figure 1C,D). Additional to these parameters, chlorophyll a and chlorophyll b content was determined by spectrophotometric measurements (Figure 1E). At the first day of flooding stress, the chlorophyll b concentration decreased about 17.2% after four hours, if compared to chlorophyll a. At the second day, chlorophyll b content was decreased about 24.3% compared to chlorophyll a. At the third day, chlorophyll b content was lowered about 17.8% in comparison to the chlorophyll a content.

Maize plants showed typical phenotypes after three days of waterlogging (Figure 1). Growth of the shoots was compared for control and flooded plants for 4, 28 and 52 h (three days of flooding). The ratio of the average shoot growth of stressed and control plants did not change significantly. Shoot growth was only slightly decreased for water logged plants after 52 h (Figure 1B). These observations confirm published data for water logged maize [27]. Reduced elongation growth is based on the negative effect of flooding on photosynthesis and is in accordance with the low oxygen quiescence syndrome of maize [9,10,27].

**Figure 2:**
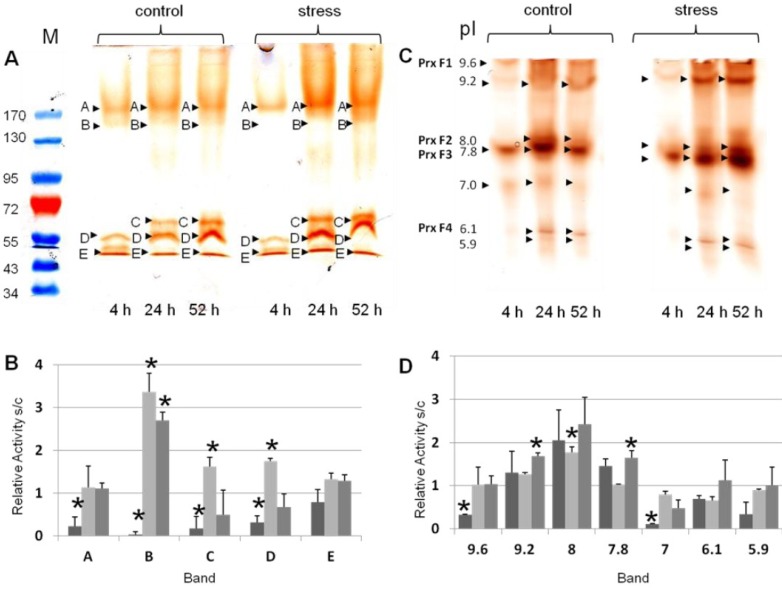
First dimension gel electrophoresis and guaiacol/H_2_O_2_ staining (**a**) Guaiacol/H_2_O_2_ staining after separation by modified SDS-PAGE. The pre-stained marker is shown on the left, indicated with M at the top of the gel. Significantly detected guaiacol bands were amounted with the letters of A–E, referring to their mass; (**b**) Relative activity of the significantly detected bands A–E in the modified SDS-PAGE (*n* ≥ 3). The corresponding bands are indicated on the x-axis. Dark grey, s1/c1, light grey, s2/c2, middle grey, s3/c3 (s, stress, c, control, 1–3, day after stress induction); (**c**) Guaiacol/H_2_O_2_ staining after separation by native isoelectric focusing polyacrylamide gel electrophoresis (IEF-PAGE). The picture was inverted to enhance the visibility. Next to the pI, peroxidase identifiers are indicated on the left hand; (**d**) Relative activity of the significantly detected bands with the pI of 9.6–5.9 in the native IEF (*n* ≥ 3). The corresponding bands are indicated on the x-axis. Dark grey, s1/c1, light grey, s2/c2, middle grey, s3/c3 (s, stress, c, control, 1, 4 h, 2, 28 h, 3, 52 h). For the gels, the type of sample was indicated at the top with control or stress. At the bottom of the gel, the day after stress induction was specified. Significant changes between control and the associated stressed sample were marked with an asterisk (student’s *t*-test).

The decrease of chlorophyll a/b ratio (Figure 2) was shown to be a typical reaction to flooding stress in the past [6,27]. The decrease of the chlorophyll a/b ratio seems to be a good marker as its change appears shortly after the plant is exposed to flooding stress, but validations are usually needed, because of variation in reaction to flooding stress in different species. Also, the thickening of the shoot stem diameter after a few days of flooding is in accordance with published data for maize [27]. The thickening of the basal shoot was shown to be based on the aerenchyma formation in the root cortex, which is the most studied morphological response to flooding stress [27,34]. Aerenchyma provides a continuous system of interconnected aerial spaces with a lower resistance for oxygen transport. Aerenchyma formation allows root growth and soil exploration under anaerobic conditions by oxygen transport from aerial shoots to submerged roots [27,34].

In the past, it was shown that continuous flooding over time causes a decrease in photosynthetic capacity of mesophyll cells and finally to an overall reduction of photosynthetic activity [27]. The lower photosynthetic activity is based on the lower chlorophyll content, reduced activity of carboxylation enzymes and oxidative damage of photosystem II by ROS. Low photon utilisation of flooded plants results mostly in ROS production [35]. The level of ROS, like superoxide anion radical, singlet oxygen, hydrogen peroxide and hydroxyl radical, which are highly reactive and provoke damage to various molecules, is tightly managed to protect the cells against oxidative stress. Plants contain antioxidants like ascorbate, glutathione and membrane embedded quinones (e.g., tocopherols and ubiquinone) and enzymes with ability to scavenge ROS and regenerate the antioxidants [28,30]. Peroxidases are part of this ROS scavenging mechanism, but they also play a role in cell wall loosening and reorganisation, such as needed for the formation of aerenchyma.

### 3.2. Differential Regulation of Soluble Peroxidases—1D and 2D PAGE Analysis

Peroxidases play roles in the ROS scavenging mechanism and in cell wall loosening and reorganisation. Data at hand showed alterations of peroxidase profiles, increases in abundance of specific peroxidases and increase or decrease of guaiacol peroxidase activities under waterlogging conditions. These observations were in accordance to published data [36,37].

Molecular mass of peroxidases and over all profiles were observed for control and stressed plants for all three time points. Four guaiacol peroxidase bands were detected in all samples after separation by modified SDS-PAGE (Figure 2A; band A, B, D, E). The peroxidase profiles were different for the observed time points (Figure 2A). From the relative stress to control (s/c) ratios, relative abundance change was calculated for the bands of the modified SDS-PAGE (Figure 2B). The strongest regulated band of peroxidase abundance was band B, with 133 kDa (Figure 2B). This band was significantly decreased after four hours of waterlogging, whereas it was increased after 28 and 52 h of waterlogging. Furthermore, peroxidase profile of four hours waterlogging exhibited a decreased band C, independent of the flooding stress. Overall, bands A–D were significantly decreased in number after four hours of waterlogging (Figure 2B). After 28 h of waterlogging, stress intensity of bands B–D were significantly increased. At 52 h of waterlogging, only band B was significantly changed. Band E was not significantly changed at any of the observed time points. The overall amount of peroxidase activity per time point, calculated from all bands per lane from all technical/biological replicates, result in the following order: stress day 2 ≥ stress day 3 ≥ control 3 ≥ control 2 ≥ control 1 ≥ stress day 1.

Each lane of modified SDS-PAGE was cut into four pieces, digested and used for identification of proteins by LC-MS (Supplemental Table S1), but peroxidases were not identified. Possible explanations for the lag of peroxidase identifications are the relatively high sensitivity of the guaiacol staining in comparison to standard staining, e.g., CCB, overlay of high abundant proteins with the same molecular mass and inefficient tryptic digestion based on the nature of the non-reducing SDS-PAGE.

Similar to the modified SDS-PAGE, peroxidase bands of the first dimension native IEF-PAGE were used to obtain isoelectric points (pI) and corresponding peroxidase profiles (Figure 2C; Table 1). Overall peroxidase profiles were comparable to the profiles of the modified SDS-PAGE (Figure 2B). Samples of the first time point showed a different peroxidase profile from the samples of the two following time points, independent of the sample treatment. Semi-quantitative analysis of the activity bands was performed for the native IEF-PAGE, as described for modified SDS-PAGE (Figure 2C). Significant changes in activity were observed after four hours of waterlogging at pI 9.6 (PrxF1) and 7.0, which were both decreased in the stressed sample. At 28 h of waterlogging, only the band with the pI of 8.0 (PrxF2) was significantly changed, if the ration of stress to control was compared. The band with the pI of 9.2 was only significantly changed at 52 h after induction of waterlogging. The band with the pI of 7.8 (PrxF3) was similar to the band with pI 9.2 significantly increased after 52 h in the waterlogged sample, if compared to the control (Figure 2D).

**Table 1:** Summary of peroxidase properties separated by one-dimensional gel-electrophoresis.

pI^ex^ *Native IEF*	kDa *Modified SDS-PAGE 1D*	kDa *hrCNE*
9.6 ± 0.3	183 ± 7	637 ± 7
9.2 ± 0.4	133 ± 5	330 ± 7
8.0 ± 0.2	68 ± 1	431 ± 8
7.8 ± 0.2	55 ± 1	219 ± 8
7.0 ± 0.1	47 ± 2	200 ± 9
6.1 ± 0.1		162 ± 8
		136 ± 0.6
		125 ± 2
		117 ± 1.7
		32 ± 1.7

Aside the specific regulations by flooding, peroxidases with acidic pIs showed a regulation independent of flooding on day two (Figure 2). It is possible that these peroxidases are differentially regulated depending on the developmental stage of the shoot [14]. Plants were grown in the glass house; therefore, changes in light conditions are also an option for this change, but daily measurements showed light intensity was comparable at all three time points (~1000 µmol/m^2^∙s).

In contrast to modified SDS-PAGE that allows estimation of peroxidase abundance, native PAGE allows estimation of peroxidase activities by quantification of the intensity of the guaiacol peroxidase bands [21]. As shown in Figure 2, nearly all isoenzymes increased by waterlogged conditions from time point one to three, showing an overall induction of soluble peroxidases. This was observed for both methods, modified SDS-PAGE and native IEF-PAGE, suggesting a relation between peroxidase abundance and activity. Abundance and activity are not related for all proteins, especially if proteins are activated by post-translational modifications [38].

First dimension hrCNE was used to calculate the native molecular mass of guaiacol peroxidases (Table 1). Based on the resolution and the high activity of the peroxidases (saturation of the bands), hrCNE could not be used for quantitation. Two different amounts of total protein (25 µg and 40 µg) were loaded to the gels to ensure the detection of both strong and faint bands (strong activity can cover light activity). Finally, 10 bands, ranging from 32–637 kDa, were detected (Table 1).

**Figure 3:**
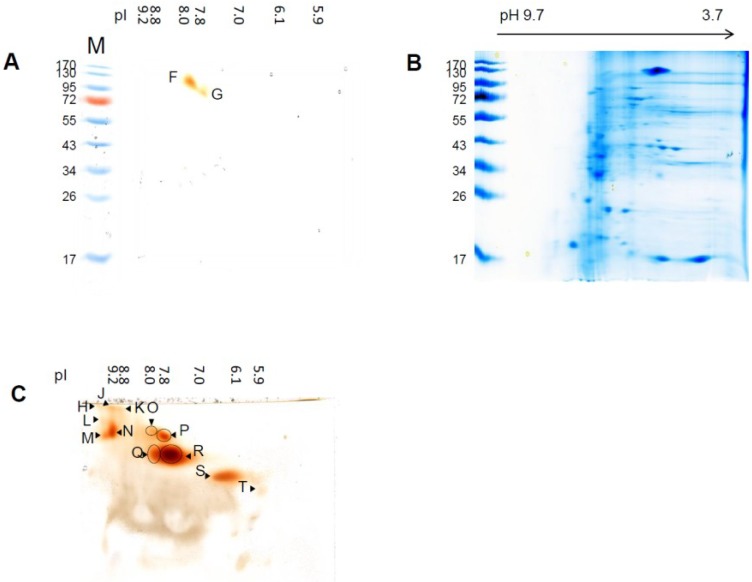
Second dimension gel electrophoresis for samples exposed to three days of waterlogged soil. (**a**) Guaiacol staining of the second dimension modified SDS-PAGE after separation by IEF-PAGE in the first dimension. The pI of the guaiacol detected spots in the first dimension was indicated at the top of the gel; (**b**) CCB staining of the gel shown in (**c**); (**c**) Guaiacol staining of the second dimension hrCNE after separation by IEF-PAGE in the first dimension. The pI of the guaiacol detected spots in the first dimension is indicated at the top of the gel.

Aside the band at 637 kDa, bands of 133 kDa and higher were detected in the modified SDS-PAGE. In both cases, molecular mass detected is fairly high for peroxidases, indicating an association with a protein complex [39,40,41]. This protein band may also present peroxidase aggregates or polymers. These results were confirmed by two dimensional gels, namely native IEF/modified SDS-PAGE and native IEF/hrCNE (Figure 3, Table 2). In the native IEF/modified SDS-PAGE combination, two peroxidases with pI 8.0 (PrxF2)/133 kDa and pI 7.8 (PrxF3)/85 kDa were detected, suggesting from their masses to be a dimer and trimer, as the identified peroxidase have an theoretical molecular mass of 27–38 kDa (Table 3). The same spots were detectable in the hrCNE with a native mass of 200 kDa.

Peroxidase profiles in the second dimension hrCNE varied for the different samples and confirmed the results of the first dimension native IEF-PAGE. The smallest amount of peroxidase spots was detectable in the samples after 4 h independent of the stress, whereas the highest amount of spots was detected after 52 h (Supplemental Figure S2). The spot with the pI at pH 8.0/7.8 (PrxF2/F3) and at pI 9.2 were clearly separated in the second dimension into two spots (pI 9.2, J, N; pI 8.0, O, Q; pI 7.8, P,R) with different native molecular masses (Figure 3C, Table 2). Spots in the second dimension were only used to get a better view on the isoenzymes with similar pI that could not be separated by native IEF-PAGE. Based on gel to gel variation, these gels were not used for quantitation, and identification by MS was not successful.

**Table 2:** Summary of peroxidase properties separated by two-dimensional gel-electrophoresis.

Spot Name	pI^ex^ *Native IEF*	kDa *Modified SDS-PAGE*	kDa *hrCNE*
H	9.8 ± 0.2	n.d.	637 ± 10
J	9.2 ± 0.1	n.d.	637 ± 6
K	8.8 ± 0.2	n.d.	637 ± 5
L	9.6 ± 0.2	n.d.	440 ± 5
M	9.6 ± 0.2	n.d.	330 ± 8
N	9.2 ± 0.1	n.d.	370 ± 7
O	8.0 ± 0.3	n.d.	370 ± 7
P	7.8 ± 0.3	n.d.	330 ± 8
F ^SDS−PAGE^/Q ^hrCNE^	8.0 ± 0.3	133 ± 8	200 ± 4
G ^SDS−PAGE^/R ^hrCNE^	7.8 ± 0.3	85 ± 4	200 ± 6
S	6.1 ± 0.1	n.d.	139 ± 5
T	5.9 ± 0.2	n.d.	115 ± 2

### 3.3. Sensitivity of Soluble Shoot Peroxidases against SDS

Second dimensions after native IEF-PAGE was separated by modified SDS-PAGE and hrCNE. Modified SDS-PAGE resulted in only two spots with a molecular mass of 133 kDa and 85 kDa and a pI of pH 8.0/7.8 (PrxF2/F3) remained active. To be sure that the protein transfer from the first to the second dimension was performed correctly, gels were stained with colloidal Coomassie Blue (CCB, Figure 3B). CCB staining proved a good transfer of proteins to the second dimension. In order to increase the potential to detect further active peroxidase spots, hrCNE was used as an alternative method for the second dimension. Using hrCNE as second dimension, it was possible to detect 12 peroxidase spots by guaiacol/H_2_O_2_ staining.

The two spots, 133 kDa and 85 kDa (PrxF2/F3), were the only detectable spots in the second dimension modified SDS-PAGE, showing that they were more stable than the other peroxidases. Spots with a comparable size were also detected in the electrophoretic analysis after separation by SEC, whereas others were not detectable anymore, also showing that these peroxidase multimers or complexes showed greater stability than others. Why these two peroxidase spots are more stable than others has to be further investigated. The identified ZmPrx66 in the band PrxF2 was earlier detected in root plasma membranes samples separated by modified SDS-PAGE [42]. However, most peroxidases investigated in the present study appeared to be sensitive against SDS. At least guaiacol staining did not work properly in second dimension modified SDS-PAGE, possibly due to loss of the heme group; even in the first dimension SDS showed no negative effect on the detection. An explanation for the greater sensitivity to SDS in the second dimension could be actually based on the fact that it was used as a second dimension and the stabilising factor was separated from the peroxidases in the first dimension, resulting in increased sensitivity.

In the past, modified SDS-PAGE was regularly used to study root membrane peroxidase abundance in the second dimension; these enzymes appeared to be more stable compared to the soluble isoperoxidases of the present study [15,20,21,23]. Henceforth, this case could be evidence for the lower stability of soluble peroxidase monomers in the shoot of maize. In any case, this result will need further investigation.

### 3.4. SEC and Identification of Peroxidases by LC-MS

To confirm the results from the gel electrophoresis, samples were separated by SEC. Peroxidase elution from the column was followed by guaiacol/H_2_O_2_ micro assay. Analysis of the different samples and biological replicates showed molecular mass from 40–287 kDa with significant variation between the separations. Furthermore, different peroxidase could not be clearly separated (Supplemental Figures S4 and S5). Additionally, active fractions were separated by one dimensional modified SDS-PAGE, native IEF and hrCNE and peroxidases detected by guaiacol/H_2_O_2_ in gel staining (Supplemental Figure S5, Table S2). Observed profiles showed strong similarities independent of the separated sample (control, stress). Molecular mass calculated for the detected bands confirmed bands from one dimensional electrophoresis separation without SEC (Table 2). Aside that, molecular mass calculated from the fraction number of the SEC varied strongly from the detected bands in the gels. Finally, only bands with a molecular mass above 120 kDa were detected after gel electrophoresis of SEC fractions, independent of the electrophoresis method. Native IEF separation was not possible for most of the samples based on a disturbed electric flow. If separation was possible, activity was detected at pI 6.1, 7.8, 8.0 and 9.6, which confirmed the primary results mentioned above. All spots detected in the native IEF were identified, after tryptic digestion, by MS as peroxidases. Experimental and theoretical properties of the identified peroxidases were summarized in Table 3, while the complete MS data set for identification of the peroxidases can be found in the Supplemental (Supplemental Table S3). The pre-separation of SEC overlaying proteins with similar pI, but different molecular mass, meant they were excluded from the separation of native IEF without diminishing the concentration of the protein. Therefore, the chance of identifying a specific protein, e.g., peroxidase, was much higher than in a first dimension modified SDS-PAGE.

In most activity spots, multiple peroxidases were identified (Table 3). With the experimental pI of the identified peroxidases (PrxF1-F4), they can be assigned to the bands found in the native IEF without pre-separation by SEC. Besides class III peroxidases, also ascorbate peroxidases (APx, class I peroxidases) were identified (ZmAPx01 and 02). These peroxidases play a major role under oxidative stress and have been shown to be regulated under stress conditions [35,43]. Even APx was identified in the bands PrxF2 and PrxF3; usually they cannot use guaiacol as substrate. In soybean, flooding stress regulated APx [22,23]. We suggest that the APxs identified do not contribute to the detected activity, which would be in accordance with earlier results. Additionally, a plasma membrane associated peroxidase (ZmPrx66) was identified in the analysed soluble fraction, which might be due to (i) a contamination or (ii) the proteins disband under specific conditions from the plasma membrane. If the second point is the case, it will have major influence on the understanding of the stress–peroxidase relation. Aside ZmPrx66, APx1 and APx2, eight more peroxidases were identified in the spots Prx F1 to Prx F4. ZmPrx06 (also named peroxidase J), ZmPrx118, ZmPrx97, ZmPrx124, ZmPrx125, ZmPrx07, ZmPrx38, ZmPrx106 were identified in the maize genome but further information on these soluble peroxidases are not known [44,45,46]. Based on KEGG (Kyoto Encyclopedia of Genes and Genomes) calculations related pathways for ZmPrx118 are the phenylpropanoid [47] and the lignin biosynthesis [48], as well as the phenylalanine metabolism [49]. ZmPrx07 was identified by the NCBI blast as ZmPrx66 precursor and showed 99% similarity to ZmPrx66, making it highly reasonable that the identified peroxidase in the spot Prx F2 is the plasma membrane associated ZmPrx66. ZmPrx42 identified in the band Prx F2 was predicted before as pmPOX3-1 [42]. In both cases, the functions discussed were removal of H_2_O_2_, oxidation of toxic reductants, biosynthesis and degradation of lignin, suberisation, auxin catabolism, response to environmental stresses such as wounding, pathogen attack and oxidative stress. These functions might be dependent on each isoenzyme/isoform in each plant tissue. Three of the identified peroxidases have been shown to be induced by biotic or abiotic stress factors (Table 3). According to the PeroxiBase, ZmPrx97, identified in band PrxF1 with the pI of 9.6, and ZmPrx66, ZmPrx42, identified in the band PrxF2 with the pI of 8.0, are induced by drought and salt stress. Our former data showed alterations of ZmPrx66 abundance at washed plasma membranes by elicitors, salicylic acid and H_2_O_2_ [25].

Class III peroxidases may build a complex functional network of different isoenzymes that appears tightly regulated under stress conditions. Depending on a stressor and plant stress responses, distinct isoperoxidases seem to be up-regulated and/or down-regulated. This was shown for maize under submerged conditions (Figure 2 and Figure 3), by different signalling compounds and by oxidative stress [25]. The observed soluble peroxidases have not been able to be assigned to a specific localisation in the cell up to now. Therefore, separation of the peroxidase function in the protective cycle or in the flooding induced leaf growth has to be further investigated. Increased lipid peroxidation and guaiacol peroxidase and APX activity have been demonstrated for maize by flooding in young maize seedlings, but resulting peroxidases were not identified [50]. Thus, ROS scavenging may be one of the major functions of guaiacol peroxidases induced under waterlogging conditions. Peroxidases may also be involved in the process of adaptation. Aerenchyma formation is correlated to programmed cell death (*i.e.*, ROS production) and cell wall stiffening. In cell wall fractions of pea (*Pisum sativum* L.) roots, alkaline isoperoxidases of ionically bound fraction appeared to be involved in elongation growth, whereas covalently bound peroxidases with acidic pI were suggested to be involved in cell wall related functions [51]. Furthermore, extracellular isoperoxidases have been demonstrated to be involved in ROS production [41,52]. ROS production has been demonstrated during root hair formation [53]. Thus, functions in formation of adventive roots may also be possible. Localisation and biochemical properties of flood-induced isoperoxidases will need further studies to clarify their physiological functions in maize.

**Table 3:** Identified peroxidases by LC-MS. Separated samples by size exclusion chromatography were followed by native IEF and stained with guaiacol/H_2_O_2_. Detected spots were tryptical digested and analysed by LC-ESI-MS/MS. Identified peroxidases and their properties are listed in the table. Detailed information about the MS results can be found in the Supplemental Table S3. Name: name used in the publication; pI exp: experimental pI resulting from the calculation of the activity band in native IEF after SEC separation; MW^exp^: experimental molecular mass (kDa), resulting from the SEC separation; Accession: accession for the peroxidase in the searched database; pI^th^: theoretical pI given by the database; MW^th^: theoretical molecular mass (kDa) given by the database; Peptides: amount of identified peptides; Class: classification of the peroxidase identified; Localisation: known cellular localisation; Inducers/Repressors: known inducers or repressors of the identified peroxidase based on information provided by peroxibase [54].

Band	pI ^exp^	MW ^exp^	Accession	Database	pI ^th^	MW ^th^	Peptides	Class	Localisation	Inducers/Repressors
Prx F1	9.6	34–51	ZmPrx06	Peroxibase	6.2	33	6	III	-	*induced by cyst nematode infection, pathogen interaction*
			ZmPrx118		5.5	37	10	III	-	-
			ZmPrx97		6.6	38	12	III	-	*induced by salt stress*
			ZmPrx124		4.7	37	15	III	-	-
			ZmPrx125		8.6	34	10	III	-	-
Prx F2	8.0	34–58	ZmPrx66	UniProt	8.0	33	2	III	PM	*induced by drought, elicitors, salicylic acid, wounding and H_2_O_2_*
			ZmPrx42	Peroxibase	5.3	33	9	III	PM	-
			ZmPrx07	Peroxibase	8.0	34	12	III	-	-
			ZmAPx01	UniProt	5.7	27	27	I	cytosolic	-
			ZmAPx02	UniProt	5.7	28	9	I	cytosolic	-
Prx F3	7.8	34–58	ZmAPx01	UniProt/Peroxibase	5.7	27	8	I	cytosolic	-
			ZmPrx38	Peroxibase	9.2	38	10	III	-	-
			ZmPrx07	Peroxibase	8.0	34	12	III	-	-
Prx F4	6.1	45	ZmPrx106	Peroxibase	8.4	34	40	III	-	-

## 4. Conclusions

In the present study, the effect of waterlogging on maize peroxidase profiles has been investigated. Isoperoxidases were altered in protein abundance, and increased guaiacol peroxidase abundance and activity was detected by proteomic approaches. The combination of native IEF-PAGE and hrCNE appears to be a powerful set-up to investigate protein adaptations under stress conditions. Second dimension modified SDS-PAGE appears problematic for most soluble guaiacol peroxidases, except for PrxF2 which was identified as ZmPrx66 amongst others, probably because of instability (e.g., lost heme groups). In the past, second dimension modified SDS-PAGE was regularly used for analysis of membrane-bound peroxidases. These peroxidases appeared to be stable under these circumstances. A recent study of our group suggests a high number of membrane bound heme-peroxidases [19] that may participate in the complex network of class III peroxidases. Thus, investigation of membrane-bound isoperoxidases will be needed to fully understand the regulatory network of peroxidases involved in abiotic and biotic stresses and other cellular mechanisms.

## Supplementary Materials

Supplementary File 1
