# Supplementary material for: Alterations in Soluble Class III Peroxidases of Maize Shoots by Flooding Stress
Source: Proteomes. 2014 Jun 26;2(3):303–22. doi: 10.3390/proteomes2030303 (PMC5302756; doi:10.3390/proteomes2030303)
Supplement: Supplementary File 1 [file proteomes-02-00303-s001.zip › proteomes-54425-supplementary-final/proteomes-54425-supplementary-final.pdf]

Supplementary Material for

Alterations in Soluble Class III Peroxidases of Maize Shoots by Flooding Stress

Claudia-Nicole Meisrimler [1], Friedrich Buck [2] and Sabine Lüthje [1,*]

[1] University of Hamburg, Biocenter Klein Flottbek and Botanical Garden, Oxidative Stress and Plant Proteomics Group, Ohnhorststraße 18, D-22609 Hamburg, Germany; E-Mail: c_m_2406@yahoo.de

[2] University Hospital Eppendorf, Institute of Clinical Chemistry, Campus Science, Martinistraße 52, D-20246 Hamburg, Germany; E-Mail: buck@uke.de

* Author to whom correspondence should be addressed; E-Mail: s.luthje@botanik.uni-hamburg.de; Tel.: +49-40-42816-340; Fax: +49-40-42816-254.

Figure S1. Modified SDS-PAGE. 1D modified SDS-PAGE (12%) used for analysis of the samples. Pictures were used to calculate the non-reduced molecular mass of the guaiacol peroxidases. Additionally to the guaiacol staining, CCB staining was done to estimate separation of the overall proteins. Gel was cut into five pieces (a, b, c, d) and analyzed by MS. Complete table with the results for the MS are shown in the Supplemental Table S1.

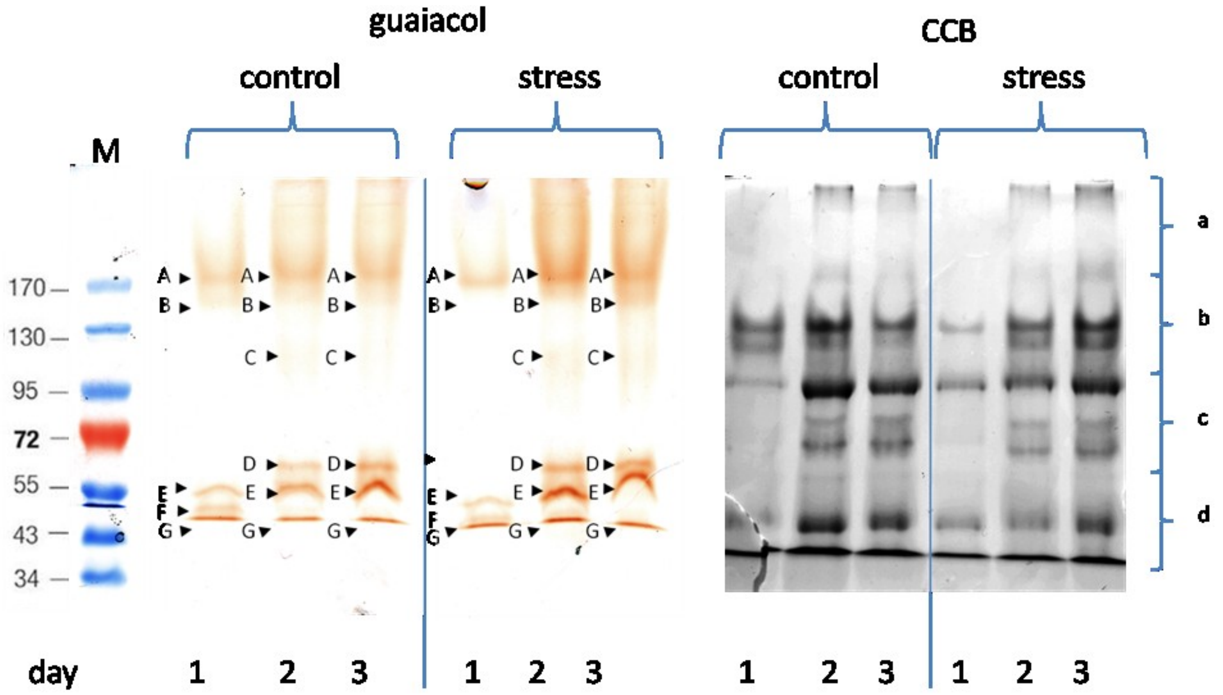

Figure S2. Native IEF/hrCNE. Typical technical examples for guaiacol/H_2O_2 staining after separation by native IEF-PAGE (4% ampholytes pH 3–10)/hrCNE (6%–15%) are shown. After 5 min of activity staining gels were scanned for documentation. Separated sample is indicated on the left.

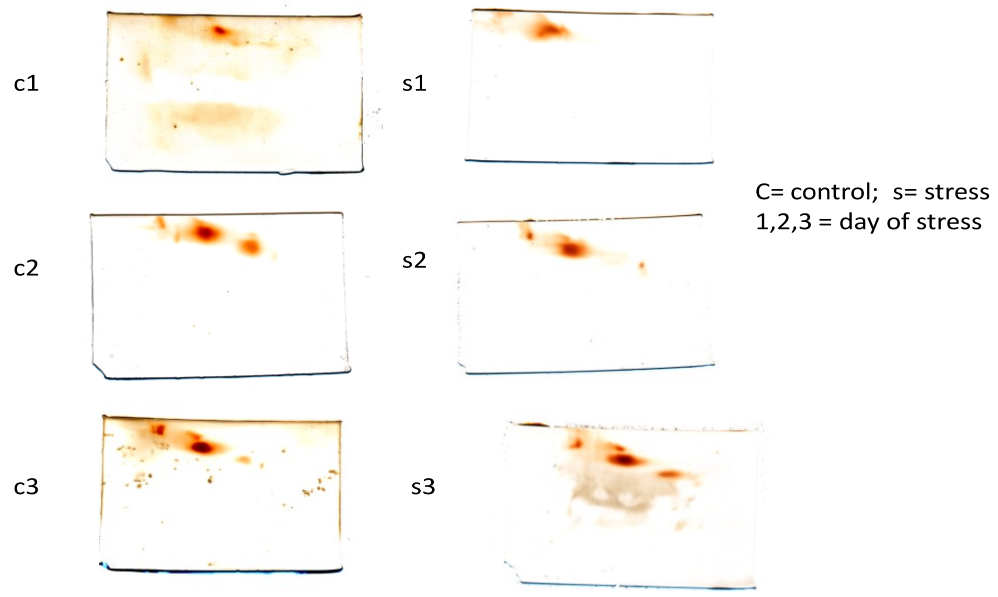

Figure S3. First dimension hrCNE. First dimension hrCNE (gradient: 4%–16%) was used for native analysis of the samples. Pictures were used to calculate the native molecular mass of the guaiacol peroxidases. (**A**) Guaiacol staining; (**B**) CCB staining; (**C**) over lay.

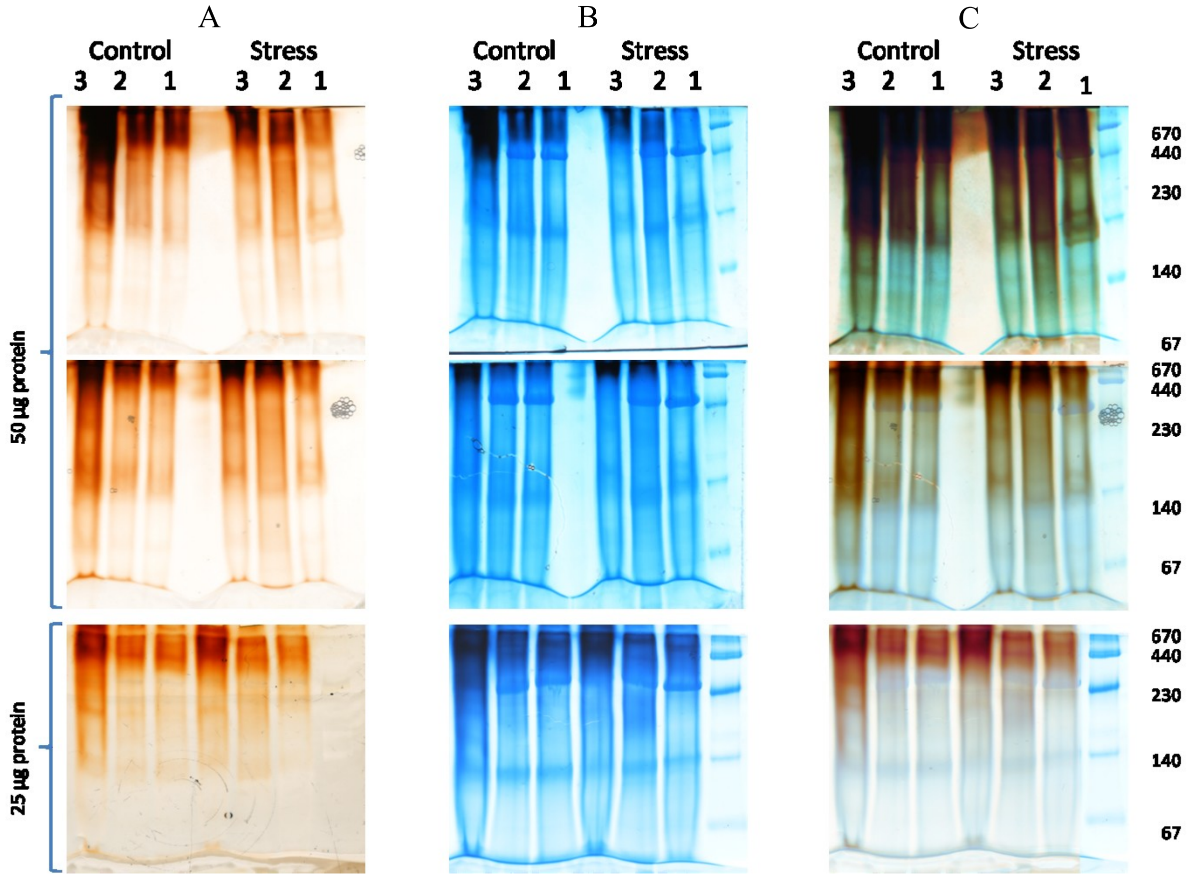

Figure S4. Elution profiles of guaiacol peroxidases separated by size exclusion (SEC): Specific guaiacol peroxidase elution profiles were tested by micro-assays using guaiacol and H_2O_2 as substrates. On the next two pages the three technical replicates for the SEC separation are presented. Each technical replicate is similar to a biological replicate.

Elution of control (C) samples 1–3(3 technical/biological replicates)

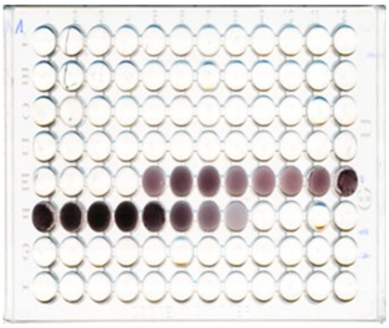

C1-1: fraction 53-68

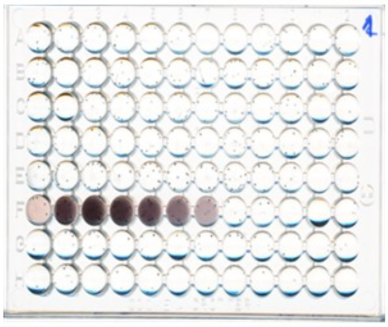

C1-2: fraction 61-68

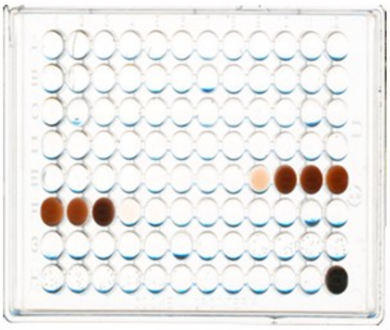

C1-3: fraction 56-63

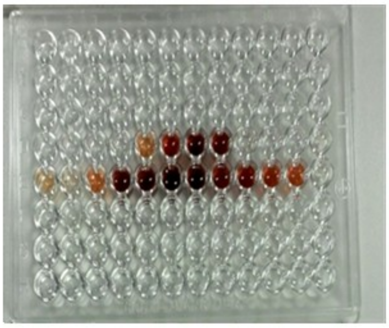

C2-1: fraction 41-44, 49, 51-59

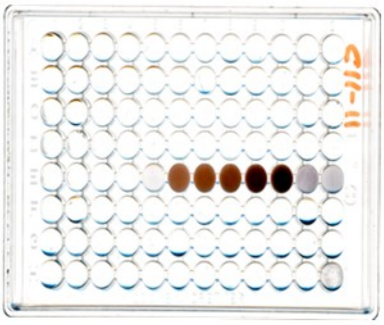

C2-2: fraction 54-60

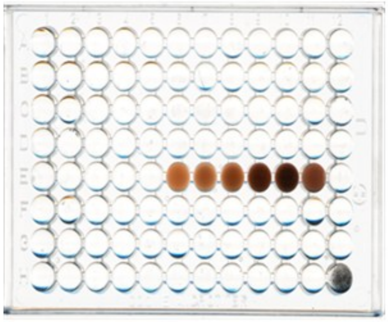

C2-3: fraction 54-59

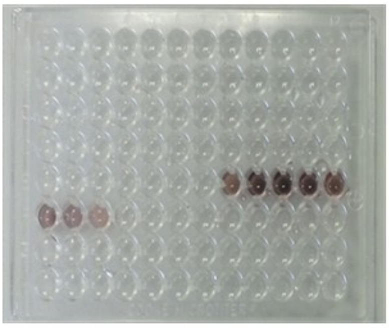

C3-1: fraction 56-63

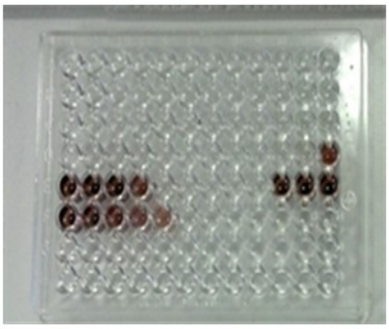

C3-2: fraction 48-52, 58-65

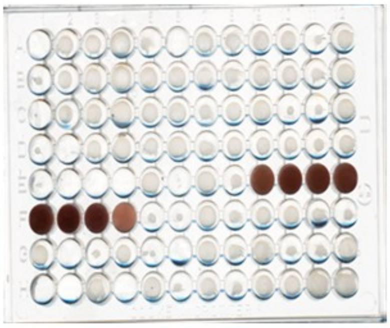

C3-3: fraction 57-64

Figure S4. *Cont.*

Elution of stress (S) samples 1–3 (3 technical/biological replicates)

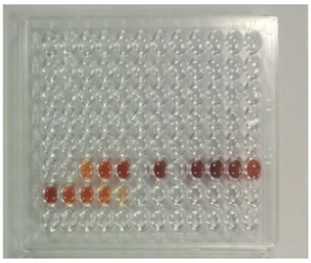
S1-1: fraction 53-55, 57, 59-67

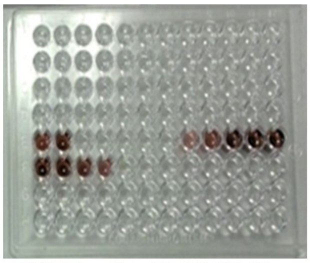
S1-2: fraction 48-49, 56-64

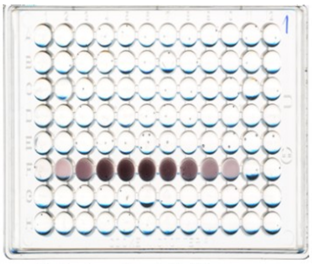
S1-3: fraction 53-61

volume shift of 6 mL, because of problems with the fractionator in sample S1-1 and S1-3, fractions would be one lane higher - already taken in account when the fractions were calculated

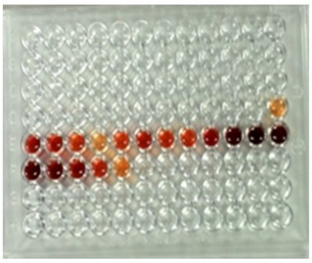
S2-1: fraction 48-65

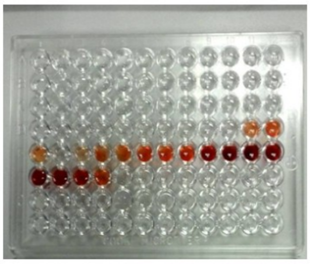
S 2-2: fraction 47-64

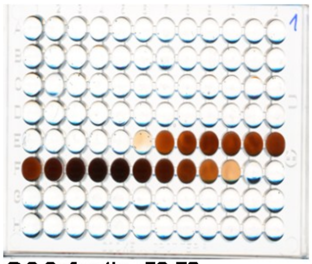
S 2-3: fraction 53-70

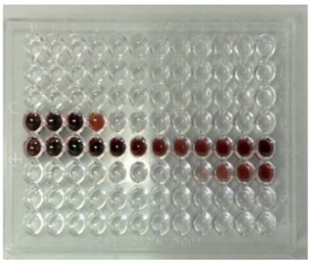
S3-1: fraction 37-40, 49-60, 69-72

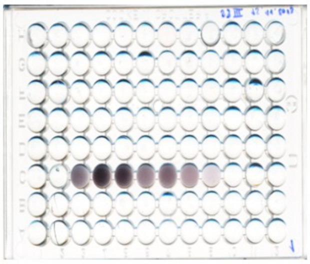
S 3-2: fraction 53-60

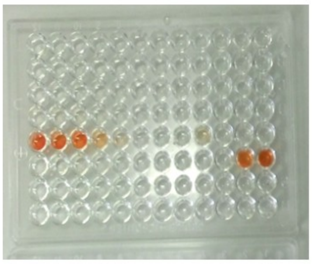
S3-3: fraction 37-41,45, 59-60

volume shift of 6 mL, because of problems with the fractionator in sample S3-2, fractions would be one lane higher - already taken in account when the fractions were calculated

Figure S5. Analysis of active SEC fractions by electrophoresis. Fractions, resulting from the separation by SEC, tested positive for guaiacol activity in the micro assay were analyzed using hrCNE (12% acrylamide concentration), modified SDS-PAGE (12% acrylamide concentration) and native IEF-PAGE (5% acrylamide concentration, pH 3–10). For each active fraction 25 µL were mixed with the PAGE corresponding sample buffer and loaded on the gel.

Analysis of sample C1 (control sample 1)

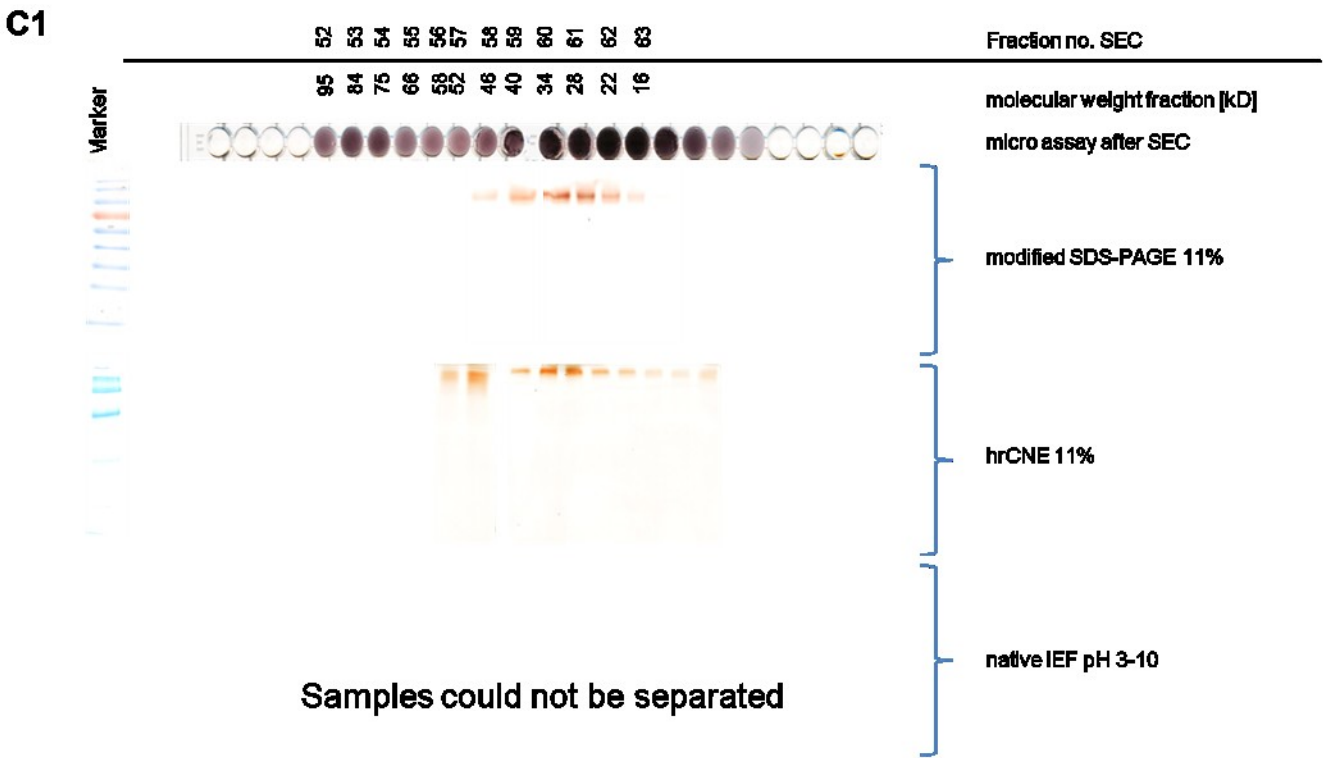

Analysis of sample C2 (control sample 2)

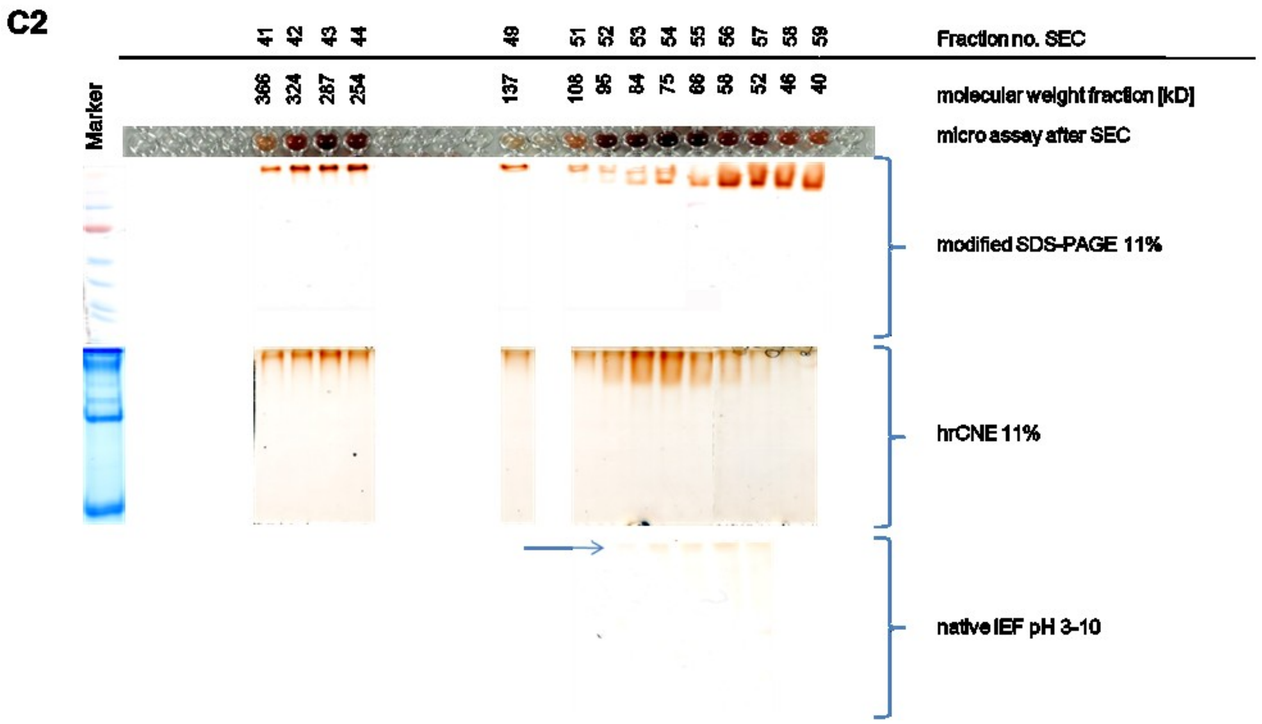

Figure S5. *Cont.*

Analysis of sample C3 (control sample 3)

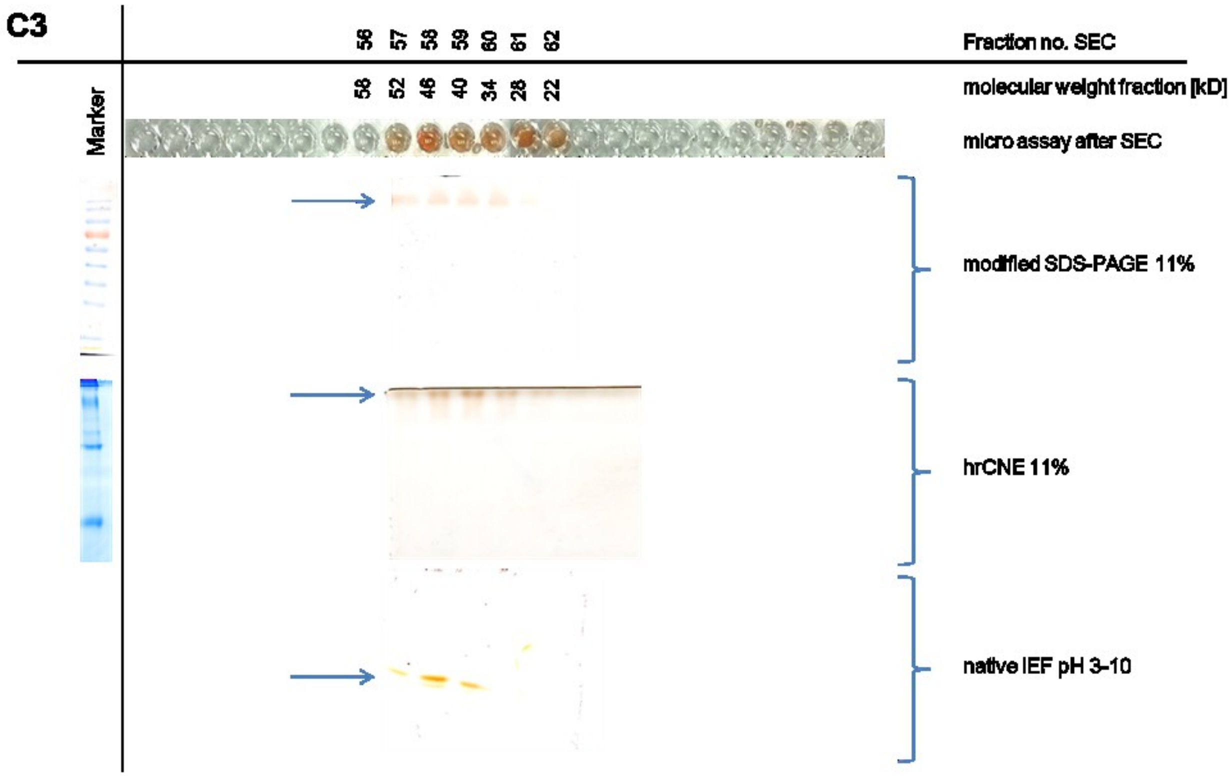

Analysis of sample S1 (stress sample 1)

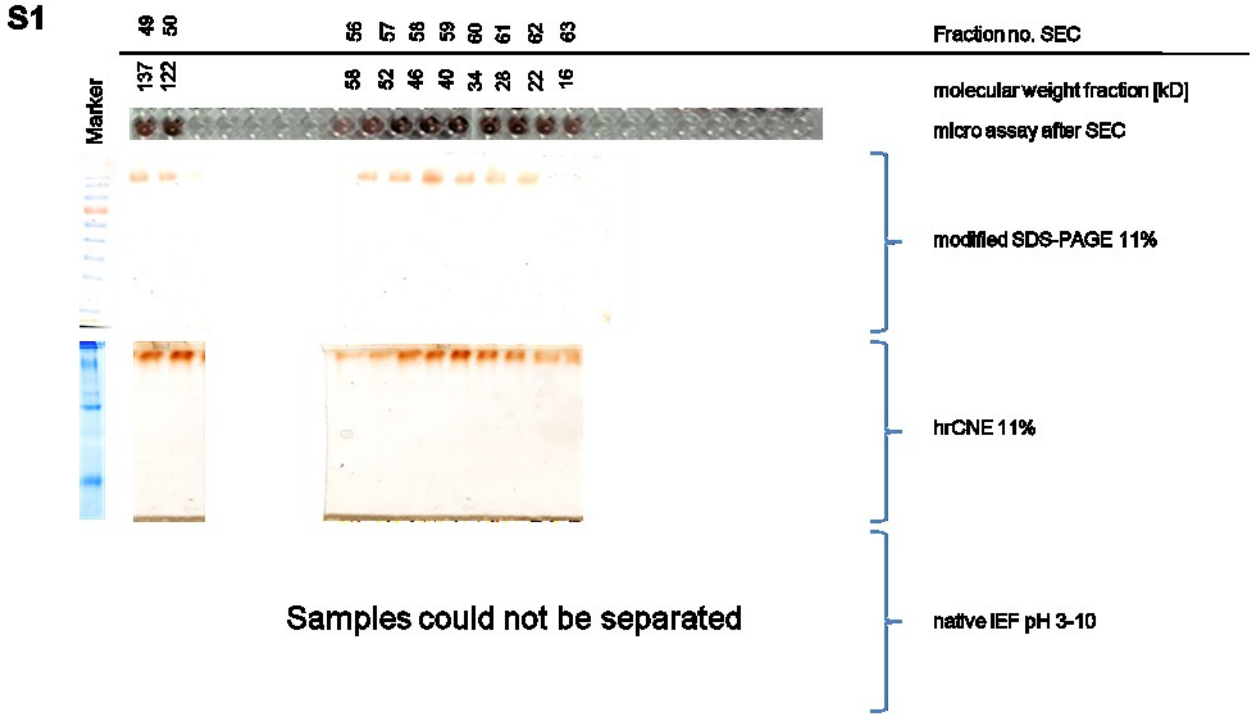

Figure S5. *Cont.*

Analysis of sample S2 (stress sample 2)

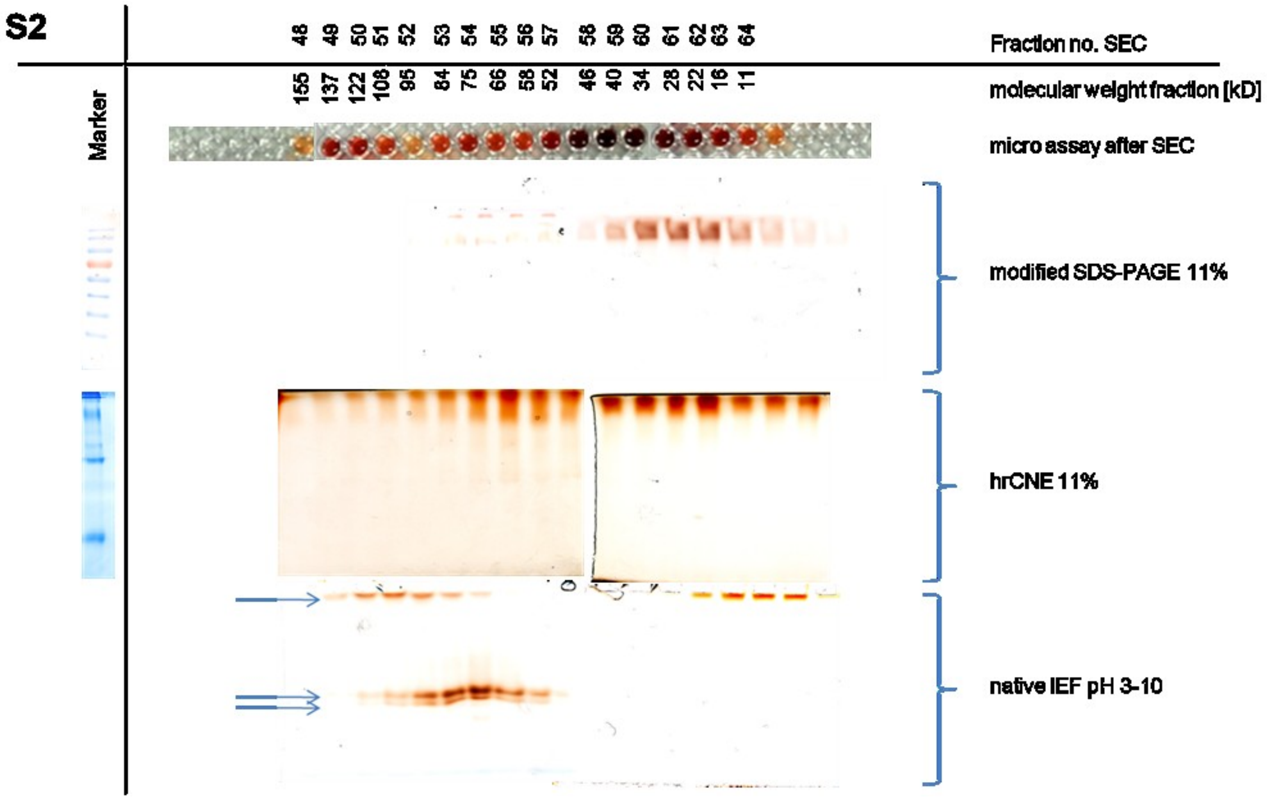

Analysis of sample S3 (stress sample 3)

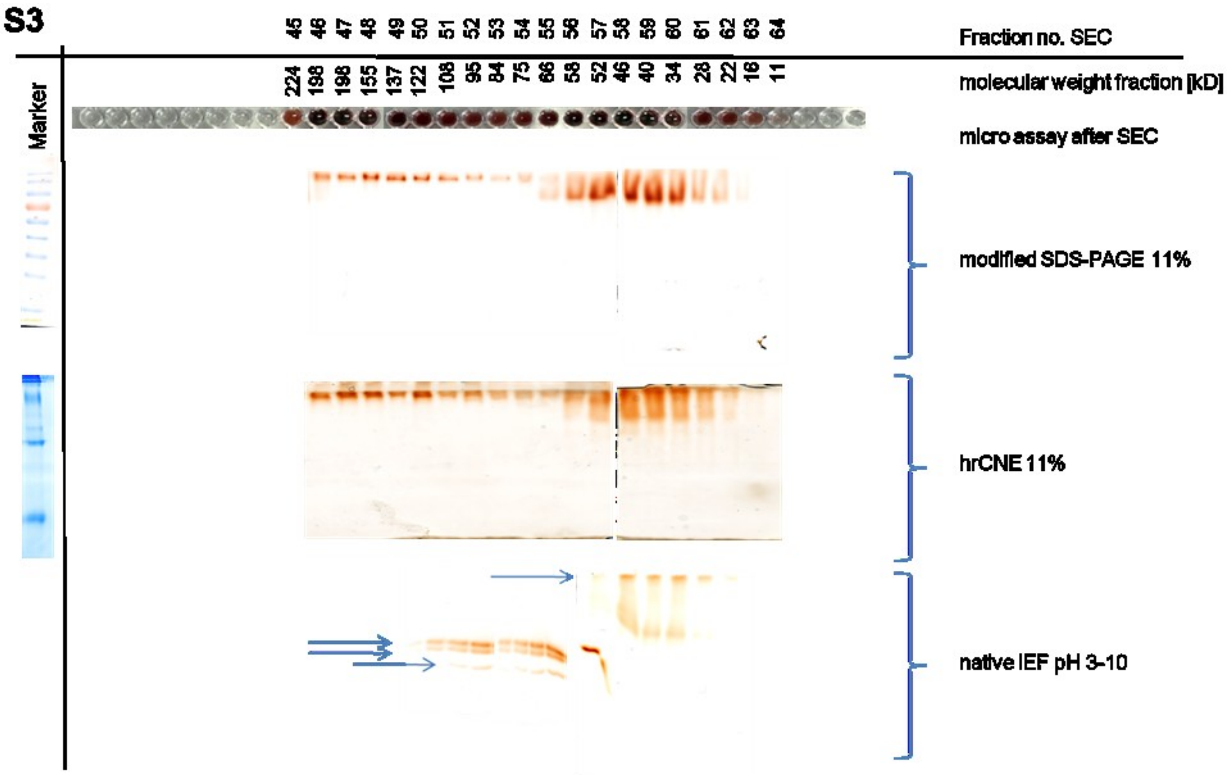

Table S2. Summarizing table for the SEC fraction analysis. Results of the data of Figure S4 and S5 were summarized in this Table. Molecular weights and pI resulting from the electrophoretic analysis of the main activity peak are shown in column three to five.

**Sample**	**kDa SEC**	**kDa modified SDS-PAGE**	**kDa hrCNE**	**pI native IEF**
		First dimension		
C1	24.5	165	310	n.d.
C2	51	157, 147, 126	406, 301	9.6
C3	58	133	410	8.0, 7.8
S1	51	146	406, 301	n.d.
S2	34	160, 124	406	9.6, 8.0, 7.8
S3	45	125	395, 230	9.6, 8.0, 7.8, 6.1
