# Supplementary figures and images for: Alterations in Soluble Class III Peroxidases of Maize Shoots by Flooding Stress
Source: Proteomes. 2014 Jun 26;2(3):303–22. doi: 10.3390/proteomes2030303 (PMC5302756; doi:10.3390/proteomes2030303)

## Guaiacol Peroxidase

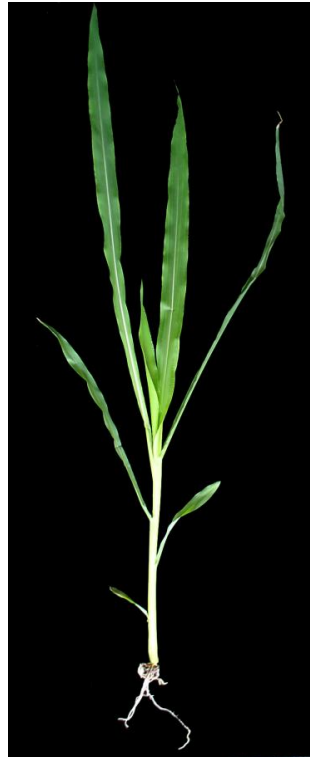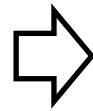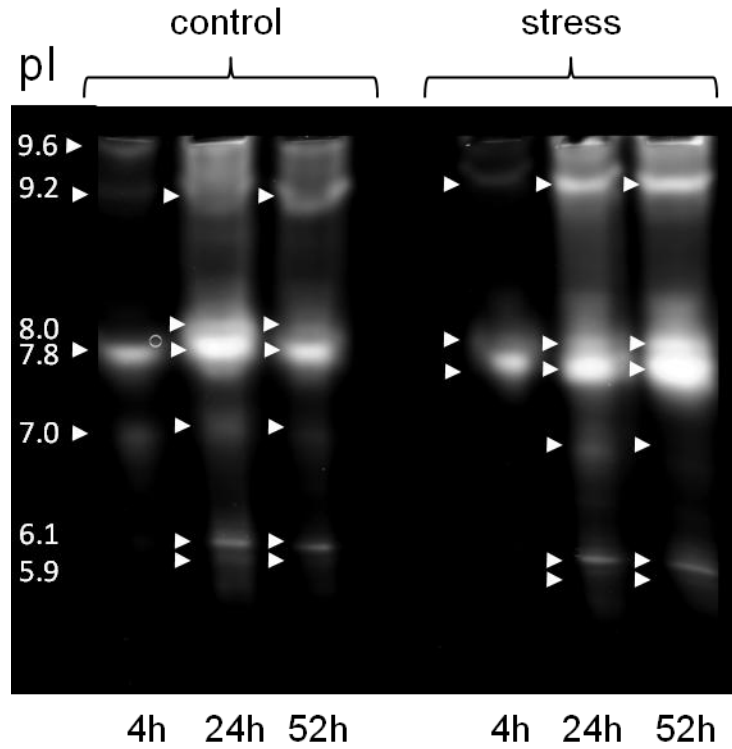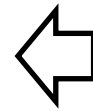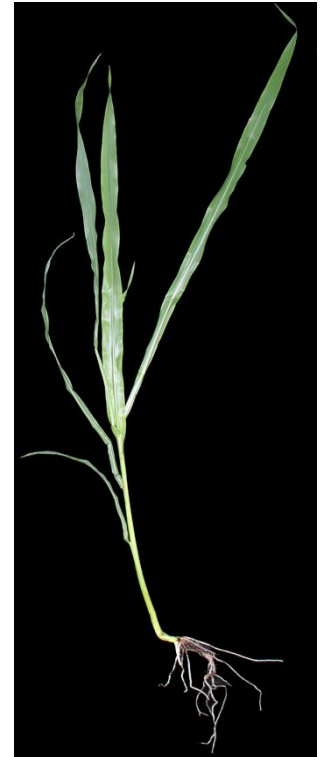

Supplement: Supplementary File 1 [file proteomes-02-00303-s001.zip › proteomes-54425-supplementary-final/proteomes-54425-GA.pdf]
